# Th17 Response and Inflammatory Autoimmune Diseases

**DOI:** 10.1155/2012/819467

**Published:** 2011-11-15

**Authors:** Janelle C. Waite, Dimitris Skokos

**Affiliations:** Department of Immunity and Inflammation, Regeneron Pharmaceuticals, Inc., 777 Old Saw Mill River Road, Tarrytown, NY 10591, USA

## Abstract

The proinflammatory activity of T helper 17 (Th17) cells can be beneficial to the host during infection. However, uncontrolled or inappropriate Th17 activation has been linked to several autoimmune and autoinflammatory pathologies. Indeed, preclinical and clinical data show that Th17 cells are associated with several autoimmune diseases such as arthritis, multiple sclerosis, psoriasis, and lupus. Furthermore, targeting the interleukin-17 (IL-17) pathway has attenuated disease severity in preclinical models of autoimmune diseases. Interestingly, a recent report brings to light a potential role for Th17 cells in the autoinflammatory disorder adult-onset Still's disease (AOSD). Whether Th17 cells are the cause or are directly involved in AOSD remains to be shown. In this paper, we discuss the biology of Th17 cells, their role in autoimmune disease development, and in AOSD in particular, as well as the growing interest of the pharmaceutical industry in their use as therapeutic targets.

## 1. Th17 Cell Differentiation

**Figure 1:**
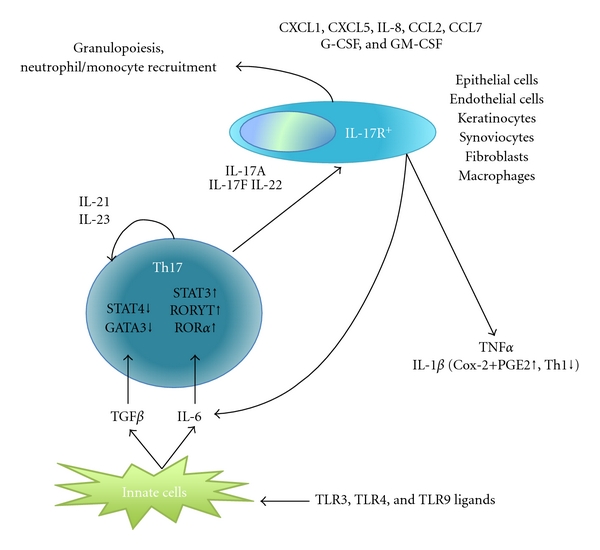
Implications of Th17 differentiation in the development of the immune response.

Th17 cells are a novel class of helper CD4^+^ T cells described in 2005 that secrete IL-17A, IL-17F, IL-21, and IL-22 [1–3]. Differentiation of naïve T cells towards a Th17 phenotype is supported by several cytokines including transforming growth factor-*β* (TGF-*β*),IL-1*β*, IL-6, IL-21, and IL-23 in mice and humans [4–8]. Indeed, ligation of toll-like receptors TLR3, TLR4, or TLR9 induces secretion of TGF-*β* and IL-6 that subsequently supports *de novo* differentiation of naïve CD4^+^ T cells to Th17, cells *in vitro* [9]. Further, it has been shown that TGF*β* is required for the initiation of Th17 dependent autoimmune encephalitis *in vivo* [10]. TGF-*β* prevents Th1 and Th2 differentiation by suppressing Stat4 and GATA-3 expression, thus allowing Th17 differentiation; however, Th17 development also occurs in the absence of TGF-*β* signaling [11, 12]. In an elegant study, Ivanov et al. demonstrated that TGF*β* and IL-6 promote IL-21R and IL-23R expression by a mechanism implicating ROR*γ*T [13]. Moreover, IL-21 expression by Th17 cells acts in an autocrine manner to promote Th17 differentiation [14, 15], while increased expression of IL-23R expression downstream of T-cell activation and Th17 development allows IL-23 signaling to further maintain Th17 activity in a Stat3-dependent manner [4, 16]. Furthermore, IL-18 synergizes with IL-23 to promote IL-17 production by IL-23-primed CD4^+^ T cells [17]. IL-18 was also shown to induce the *ex vivo* release of IL-17 and IL-23 from lymphocytes of systemic lupus erythematosus (SLE) patients [18]. It is well established that IL-18 is an important cytokine implicated in promoting Th1 polarization, which is characterized by interferon (IFN-*γ*) release. IFN-*γ* secretion subsequently activates both neutrophils and macrophages, resulting in the intracellular killing of bacteria and fungi [19]. In contrast, IL-1*β* negatively regulates Th1 differentiation by inducing cyclooxygenase (COX)-2, which subsequently increases the secretion of prostaglandin E2 (PGE2) that suppresses IFN-*γ*  production [20] thus allowing Th17 differentiation. Indeed, IL-1*β* has been shown to enhance Th17 responses in the presence of IL-23 [4, 7]. While further investigation is needed to better understand the biology of Th17 differentiation and the parameters involved in this process, overall the above studies shed light and reveal the complexity of this pathway (summarized in Figure 1).

## 2. IL-17: Expression and Function

IL-17 is a potent proinflammatory cytokine that amplifies ongoing inflammation by inducing expression of tumor necrosis factor-*α* (TNF-*α*), IL-1*β*, and IL-6 in epithelial and endothelial cells as well as other cell types such as keratinocytes, synoviocytes, fibroblasts, and macrophages. IL-17, also known as IL-17A, is the founding member of the structurally related IL-17 family, which includes IL-17B, IL-17C, IL-17D, IL-17E (also called IL-25), and IL-17F. IL-17F shares the highest amino acid identity with IL-17A [21]. The IL-17 receptor family also has five members with conserved extracellular fibronectin III-like domains and cytoplasmic “similar expression to FGF/IL-17R” (SEFIR) domains [22]. IL-17 receptor expression profiles show that IL-17RA and IL-17RC are present in different cell types and tissues. Immune cells mostly express IL-17RA, while IL-17RC is found on the surface of epithelial cells and fibroblasts [23, 24]. Upon engagement, IL-17R signaling activates NF-*κ*B, MAPK, and C/EBP pathways via SEFIR domain containing the adaptor protein Act1. Act1 further interacts with TRAF6 and TAK1 to promote signal transduction and cytokine and chemokine secretion [25]. Indeed, IL-17 stimulates production of chemokines such as CXCL1, CXCL5, IL-8, CCL2, and CCL7 that are responsible for recruitment of neutrophils to the site of inflammation [26]. For example, local production of IL-17 by Th17^+^ cells in the central nervous system (CNS) promotes neutrophil recruitment in a mouse model of multiple sclerosis (MS), experimental autoimmune encephalomyelitis (EAE) [3, 10]. Furthermore, IL-17 enhances granulopoiesis by triggering expression of G-CSF and GM-CSF [27–29]. Although GM-CSF expression is not required for Th17 differentiation, a recent study showed that this cytokine is required for autoimmune neuroinflammation via myeloid cells (CD45^hi^, CD11b^+^) infiltrating the central nervous system during the effector phase of the response [30, 31].

## 3. Th17 Cells and Microbial Immunity

Th17 cells play an important role in host defense against bacterial and fungal infection, especially at mucosal surfaces. Production of IL-17 and IL-22 upon Th17 cell triggering improves mucosal barrier surfaces by stimulating release of antimicrobial peptides and recruiting neutrophils. However, in viral and parasitic infections, Th17 responses can be detrimental to the host. During Theiler's murine encephalomyelitis virus (TMEV) infection, anti-IL-17 treatment inhibits viral persistence and development of demyelinating disease [32]. Further, it was demonstrated in this study that *in vitro* treatment of bone marrow (BM) cells or astrocytes with recombinant IL-17 increases expression of antiapoptotic molecules Bcl-xl and Bcl-2, thus preventing destruction by cytotoxic T cells. This results in persistence of the infection by enhancing the survival of virus-infected cells. In addition, it has been shown that pretreatment of target cells (coated with viral peptide) with IL-17 reduced their susceptibility to cytolytic killing by CD8^+^ T cells. In contrast, pretreatment of CD8^+^ T cells does not affect cytotoxicity. These results indicate that IL-17 alters the susceptibility of the target cell to apoptosis rather than the effector T cell's ability to kill infected cells. *In vivo* experiments using TMEV-infected mice treated with anti-IL-17 antibody showed that IL-17 blockade enhances viral load and reduces expression of prosurvival Bcl-xl and Bcl-2 proteins in both infiltrating macrophages and resident microglia. This antibody treatment significantly increases the elimination of newly introduced viral epitope peptide-loaded target cells. Altogether, these findings suggest that IL-17 expression promotes viral persistence by inhibiting apoptosis of virally infected cells. 

Moreover, in parasitic infection, IL-27R-deficient mice infected with Toxoplasma gondii develop severe neuroinflammation that is characterized by prominent IL-17 response [33]. When infected with Leishmania major, mice lacking IL-27R develop more severe lesions that are associated with the presence of IL-17^+^ CD4^+^ cells [34]. Overall, these experiments suggest that the inappropriate development of Th17 cells in some infections could lead to increased disease progression and chronic infection rather than remission and microbe clearance.

## 4. Th17 Cells in Preclinical Models of Autoimmunity

### 4.1. Rheumatoid Arthritis (RA)

Several mouse models exist for studying the mechanism of RA development. In these models, genetic inactivation of IL-17A or IL-17F has shown that Th17 cells do play a role in disease progression (summarized in Table 1). 

In the collagen-induced arthritis (CIA) model, injection of type II collagen (CII) together with Complete Freud's adjuvant (CFA) containing heat-killed *Mycobacterium tuberculosis *triggers cell-mediated and humoral responses characterized by cellular infiltration and synovitis of the joints, resulting in swelling of the paws and progressive destruction of bone and cartilage. Disease is detected by measuring CII-specific antibody levels in the serum. It has been shown that mice deficient in IL-23 (p19 subunit) show reduced numbers of IL-17^+^ cells in the draining lymph nodes and have less severe disease than mice lacking IL-12 (p35 subunit) [35]. In the same model, disease incidence and severity are greatly attenuated in IL-17A KO mice [36]. Further, IL-17A and IL-17F double KO mice do not show additional disease suppression, indicating that IL-17F does not have any additive or synergistic effect [23]. These data suggest that IL-23-dependent Th17 function and production of IL-17 support arthritis disease development.

Mice with a mutation in the gp130 subunit of IL-6R (Y759F) have disrupted SOCS3-mediated negative feedback and develop spontaneous arthritis as assessed by degree of swelling in the joints. In this model, increased IL-6 expression via hydrodynamics-based transfection enhances disease progression in an IL-17A-dependent manner [37]. IL-17A-deficient mice were resistant to arthritis while elevated IL-17 expression induced arthritis. Interestingly, IL-6 was required for IL-17A-induced arthritis, in Y759F mutants. These findings suggest that a positive feedback loop of IL-6 and IL-17 secretion is active in arthritis. 

Mice with an activating mutation in the SH2 domain of ZAP-70, a key signal transduction molecule in T cells, spontaneously develop T-cell-mediated chronic autoimmune arthritis [38]. These so-called SKG mice develop autoreactive T cells due to defective negative selection in the thymus. In this model, arthritis is suppressed in the absence of IL-17A [39], suggesting that the pathogenic T cells are indeed Th17^+^ cells. 

Further, transgenic mice carrying the human T-cell leukemia virus type I (HTLV-1) tax gene with its own LTR promoter (HTLV-I Tg mice) develop chronic inflammatory polyarthropathy that resembles RA in humans [40]. This model is dependent on IL-1*α*/*β* and IL-6 and was recently shown to be dependent on IL-17A, as IL-17A KO mice are resistant to arthritis [41].

### 4.2. Inflammatory Bowel Disease and Crohn's Disease

The roles of Th17 cells in inflammatory bowel disease (IBD) and Crohn's Disease (CD) are somewhat more controversial than in other autoimmune diseases. Th17 cells were studied in a model for IBD, where naïve CD4^+^CD45RB^hi^ cells adoptively transferred to lymphopenic mice cause colitis characterized by weight loss, diarrhea, and histological analysis (degree of epithelial hyperplasia and goblet cell depletion, leukocyte infiltration in the lamina propria, area of tissue affected, crypt abscesses, submucosal inflammation, and ulcers). Indeed, IL-17A KO CD4^+^ T cells accelerated T-cell-mediated intestinal damage compared to wild-type (WT) CD4^+^ T cells [42]. IL-17A KO T cells displayed increased IFN*γ* production, suggesting that IL-17A can downregulate Th1 response and potentially protect against Th1-mediated tissue damage. However, this finding is controversial as another group using the same model showed that IL-17A, IL-17F, or IL-22 KO T cells induced colitis equally compared to WT T cells [43]. Treatment of mice upon adoptive transfer of IL-17F KO T cells with a neutralizing anti-IL-17A antibody significantly suppressed disease, indicating that IL-17A and IL-17F have redundant biological effects. 

Additional models of IBD used to investigate the role of Th17 cells give mixed results as well. In the dextran-sodium-sulfate- (DSS-) induced colitis model, in which disease progression is primarily due to increased neutrophilic infiltration of the inflamed tissue, mice lacking IL-17F or IL-17A or mice treated with an anti-IL-17A antibody show severe weight loss and colonic epithelial damage [44, 45]. Conversely, a different study reported that genetic inactivation of IL-17A substantially reduces colitis based on both clinical score and mortality compared to WT animals, suggesting that IL-17 promotes colitis [46]. In this study, G-CSF and MCP-1 are reduced in DSS-treated IL-17A KO mice compared to treated WT mice. The disparities in these studies may be due to different intestinal microbial flora that affects the immune response in this disease model. Interestingly, IL-22 expression by CD4^+^ T cells and NK cells also has a protective role in both the DSS and T cell transfer models, as transfer of T cells from IL-22-deficient mice worsens disease [47].

It has recently been shown that during intestinal inflammation induced by anti-CD3 treatment, pathogenic Th17 cells in the gut express IL-10 receptor (IL-10R) and are negatively regulated by Foxp3^−^ and Foxp3^+^ Tregs in an IL-10-dependent manner [48]. IL-10R expression is required for Tregs to maintain sufficient IL-10 production and mediate inhibition of Th17 cells [49]. Paradoxically, Treg depletion in Foxp3-DTR mice may result in a reduced frequency of antigen-specific IL-17 producers in draining lymph nodes and blood and is correlated with reduced inflammatory skin responses during *Candida albicans* infection. This is likely mediated by a regulation of IL-2 availability [50, 51].

### 4.3. Psoriasis

IL-17^+^ T cells are increased in the dermis of psoriatic skin lesions [52]. In mice, topical application of imiquimod (IMQ), a TLR7/8 ligand, induces psoriasis-like dermatitis characterized by increased epidermal cell proliferation, neutrophil accumulation, and CD4^+^ T cell infiltration [53]. In this model, IMQ induces IL-23, IL-17A, and IL-17E expression in the epidermis and increased Th17 cells in the spleen. Furthermore, dermatitis is almost completely blocked in mice deficient in IL-23R and IL-17R. Psoriasis-like epidermal hyperplasia can be induced in the ears of mice by directly injecting IL-23 [54] and was shown to be IL-6 dependent [55]. In IL-23 induced psoriasis, CCR6^+^Th17^+^ cells accumulate in psoriatic skin where CCL20 (the CCR6 ligand) expression is abundant. Comparison of CCR6 KO and WT mice confirmed that CCR6 expression was required for IL-23-induced dermatitis. IL-22 and IL-17A are also necessary for epidermal hyperplasia in this model, as dermatitis was greatly reduced in IL-22 KO or IL-17A KO mice [56]. Further, K5.Stat3C transgenic mice constitutively express activated Stat3 within keratinocytes and develop skin lesions with histological and cytokine profiles similar to those seen in human psoriasis [57]. In this model, anti-IL-23 antibody treatment blocks epidermal hyperplasia and lowers transcript levels of Th17 cytokines (IL-17 and IL-22), *β*-defensins, and S100A. However, blocking IL-17 with anti-IL-17 antibodies or using genetic inactivation models only moderately reduced psoriasis. Overall, these findings show that IL-23-mediated induction of IL-17 secretion and Th17^+^ recruitment in the inflamed tissue efficiently promote dermatitis; however, IL-17 itself is only partially required. Interestingly, in this model, the T-cell-independent psoriatic dermatitis that spontaneously develops in IL-1RN KO mice is not affected by IL-17A deficiency [58].

### 4.4. Type 1 Diabetes (T1D)

*In vitro* activated OT1 T cells, transgenic CD8^+^ T cells that express the alpha and beta T-cell-receptor specific for OVA peptide, induce rapid diabetes onset when transferred to mice expressing OVA peptide in pancreatic beta islet cells (RIP-OVA mice). It has been shown that *in vitro* treatment of OT1 cells with IL-23 promotes their differentiation into IL-17 producing CD8^+^ T cells (Tc17) and causes diabetes in an IL-17A- and IL-17F-dependent manner when adoptively transferred to RIP-OVA mice [59]. In a different setting, using the nonobese diabetes (NOD) animal model for Type 1 diabetes, disease is inhibited following treatment with anti-IL-17A antibodies during the effector phase of disease (at 10 weeks of age) rather than during the initiation of disease (mice less than 5 weeks of age) [60]. In contrast, it has been shown that IL-17A KO on the NOD background have comparable incidence of hyperglycemia to NOD mice with a WT allele for IL-17A. This could be due to expression of other IL-17 family members such IL-17F. Thus, while Th17 cells and IL-17A seem to play a role in T1D, the precise mechanism is not yet completely understood.

## 5. Th17 Links Autoimmunity with Host Defense

While it is clear that uncontrolled inflammation causes autoimmune pathologies, it remains to be seen what triggers the inflammation in these diseases. Several new studies suggest that exposure to pathogens initiates immune responses with an autoinflammatory outcome. Indeed, Th17 cells are induced by commensal bacteria in the small intestine lamina propria and are dependent on TGF*β* but independent of IL-21, IL-23, and TLR signaling (MyD88 and Trif) [61]. However, the link between Th17 cells in the gut and autoimmune disease development at peripheral sites is not shown. 

**Figure 2:**
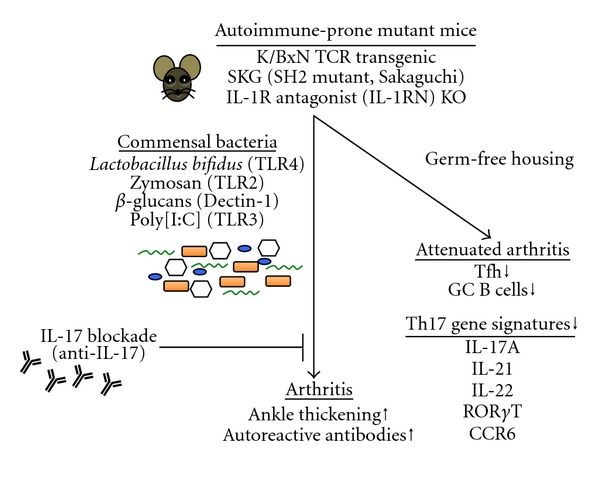
Linking Th17 cells and IL-17 to infection and autoimmunity.

K/BxN transgenic mice express autoreactive T cells specific for glucose 6 phosphate isomerase that promotes generation of high levels of auto-reactive antibodies, which subsequently induces ankle thickening. When these mice are housed in germ-free (GF) conditions, arthritis is greatly attenuated [62]. Indeed, disease reduction is correlated with a decreased number of T follicular helper cells and germinal center B cells in GF mice, suggesting that lack of microbes and their effect in adaptive immune response accounts for the reduced arthritis. Further, analysis of helper T cells from K/BxN mice in GF conditions compared to animals housed in specific pathogen-free (SPF) facility shows a significant reduction in Th17 gene signatures (IL-17A, IL-21, IL-22, ROR*γ*T, and CCR6). Neutralization of IL-17 with blocking antibodies at the time of arthritis onset (25 days old mice) completely abrogates disease, which is reflected in the low serum autoantibody titers. Moreover, following adoptive transfer, B cells from IL-17R-deficient mice failed to partake in the germinal center development. Overall, this study reveals a potential role of the IL17 pathway in regulating B cell function that a lack of Th17 cells and their downstream effects on germinal center B cells are critical factors in the disease reduction. A different study using IL-1R-antagonist- (IL-1RN-) deficient mice that spontaneously develop T-cell-mediated arthritis further supports a contribution of Th17 cells in autoimmune progression. Indeed, IL-1RN KO mice housed in germ-free conditions display reduced arthritis assayed by ankle thickness and histopathological analysis, while arthritis can be induced by infection with *Lactobacillus bifidus* in a TLR4-dependent manner [63]. Disease remission is associated with reduced Th17 cell number and decreased IL-17 secretion. In support of the above findings, mice in the SKG model (described above) do not develop arthritis if the animals are housed in microbially clean environment, despite the presence of arthritogenic autoimmune T cells, [64]. Treating mice with the yeast TLR2 ligand zymosan or glucose polymer *β*-1, 3-D-glucans (*β*-glucans), the main constituents of zymosan, induces severe arthritis in SKG mice. Blockade of Dectin-1, a major *β*-glucan receptor, is able to prevent SKG arthritis triggered by *β*-glucans. Finally, polyinosinic-polycytidylic acid (poly[I:C]), a double-stranded RNA, also showed a mild arthritogenic effect in SKG mice. Together these studies strongly support the existence of a link between microbial stimuli and innate signaling to Th17 activation and autoimmunity (summarized in Figure 2).

## 6. Potential Small Molecules to Target Th17 Cells

Recent studies have identified small molecules that may be used as therapeutics to target Th17^+^ cells. The small molecule Halofuginone selectively inhibits mouse and human Th17 differentiation by activating the amino acid starvation response (AAR), a cytoprotective signaling pathway. Indeed, treatment with Halofuginone protects mice from Th17-associated EAE [65]. Further, the cardiac glycoside Digoxin inhibits murine Th17 cell differentiation without affecting differentiation of other T-cell lineages and reduces the severity of EAE in mice [66]. In addition, leukemia inhibitory factor (LIF) produced by neural progenitor cells is able to ameliorate EAE by selectively inhibiting pathogenic Th17 cell differentiation [67, 68].

## 7. Th17 Cells in Human Pathology and FDA-Approved Drugs

In addition to preclinical results, clinical data show a correlation between enhanced IL-17 production and increased frequencies of Th17 cells in human disease (summarized in Table 2). High IL-17 levels are detected in the sera and biopsies of RA and SLE patients [18, 69–71]. *Ex vivo*, IL-17A promotes induction of proinflammatory cytokines IL-1*β* and IL-6 expression in synoviocytes from RA patients [72]. 

In MS patients, IL-17A mRNA is detected in cerebrospinal fluid mononuclear cells, and myelin reactive Th17^+^ cells are also enriched [73]. Indeed, Th17^+^ cells from MS patients produce high amounts of IL-22 and IFN*γ* and have the ability to cross the blood brain barrier, which likely contributes to the mechanism of disease pathology [74]. 

Moreover, CD8^+^ T cells secreting IL-17 and IL-22 (Tc17 and Tc22, resp.) are thought to mediate pathogenic inflammation in psoriasis. In fact, IL-17A, IL-22, and IL-23 are elevated in psoriatic skin, and IL-17A together with TNF*α* induces the expression of genes involved in psoriasis in human keratinocytes [79, 80]. Furthermore, single-nucleotide polymorphisms (SNPs) in genes involved in IL-23 signaling (IL23A, IL23R, and IL12B) are also associated with psoriasis [87]. In addition, uncommon *Il23r* variants inversely correlated with susceptibility to IBD have also been found in IBD patients [83].

A significantly higher number of IL-17^+^ cells is detected in disease-affected gut areas compared to healthy areas of the same subjects with Crohn's disease (CD) [84]. These cells express ROR*γ*T and are IL-23R^+^ and CCR6^+^. Functionally, they are capable of providing B-cell help, display low cytotoxic ability, and are resistant to Foxp3^+^ Treg suppression. Together this data demonstrates that Th17^+^ cells are indeed detected in the inflamed tissues of CD patients. An additional marker for Th17 cells in humans could be the C-type lectin-like receptor CD161, which has recently been described to promote T-cell expansion and is expressed on a discrete subset of human CD4^+^ T cells in circulation and in the gut of CD patients [85]. CD161^+^CD4^+^ T cells display an activated Th17 phenotype as indicated by increased expression of IL-23R and production of IL-17 and IL-22.

Biologic therapeutics blocking pathways implicated in Th17 development or blocking IL-17 itself are currently being developed. An anti-IL6 receptor antibody (tocilizumab, Hoffmann-La Roche and Chugai) is currently used to treat RA and Crohn's disease [76], while ongoing clinical trials are being developed by diverse biotechnology and pharmaceutical companies. While IL-6 signaling is important for Th17 development, it remains unclear if the mechanism of action of this blocking antibody is through prevention of Th17 development. 

IL-1 receptor antagonist (anakinra) has been successfully used in treatment of RA [77] and the mechanism of action could be mediated by reduction of Th17^+^ cells, although IL-1R signaling affects several immune pathways. Further studies are required to determine if Th17 cells are affected by either of these therapies.

In addition, blockade of IL-23 signaling using a monoclonal antibody (ustekinumab) against the p40 subunit of IL-12 and IL-23 has been shown to be effective in Crohn's disease, psoriasis and psoriatic arthritis with results similar to those seen when blocking IL-6 or TNF*α* [81, 82, 86]. This antibody has no substantial effect as a therapy for MS despite several preclinical studies using the EAE model in mice [75], providing additional evidence that findings generated in animal studies need to be verified using human samples in order to be validated. 

A humanized antibody blocking IL-17A is being developed to treat RA (LY2439821, Eli Lilly)[78], and phase I clinical trials showed positive results. Another humanized anti-IL-17 antibody is being developed for the treatment of RA, psoriasis, and uveitis [88]. Patients treated with this antibody show a similar reduction in symptoms to those treated with the anti-TNF blocking antibody infliximab (Remicade), validating IL-17A as a strong mediator in inflammatory autoimmune diseases.

Paradoxically, recent studies suggest that IL-17 could have a protective role in atherosclerosis [89] and that Th17 cells seem to regulate autoimmune manifestations associated with AIRE deficiency. Similarly, chronic mucocutaneous candidiasis is associated with elevated Th17 cytokines in APECED patients, further linking the Th17 pathway with autoimmunity [90].

## 8. Adult Onset Still's Disorder and Th17

Adult onset Still's disorder (AOSD) is an inflammatory autoimmune disorder characterized by high spiking fever, evanescent rash, arthritis, hepatosplenomegaly, variable multisystemic involvement, and laboratory abnormalities that include neutrophilic leukocytosis and hyperferritinaemia [91, 92]. It has been shown that proinflammatory cytokines IL-1*β*, IL-6, and IL-18 are elevated in AOSD patients [93–96]. As described above, these cytokines are associated with inducing and maintaining Th17 function. To test if Th17 cells were elevated in AOSD patients, Chen et al. performed intracellular cytokine staining and flow cytometry to assay for Th17 cells in peripheral blood. In this study, it was found that Th17 cells are significantly elevated in patients with active untreated AOSD compared to healthy volunteers. Further, the cells decrease nearly 10-fold during clinical remission and serum levels of ferritin decrease as well [97]. The frequencies of Th17 cells are similarly elevated in patients with active SLE, which was previously shown to have increased Th17 cells [98]. Furthermore, the frequency of Th17 cells was positively correlated with levels of Th17-related cytokines (IL-17, IL-1*β*, IL-6, IL-18, IL-21, and IL-23) and with disease activity score based on serum ferritin levels. Interestingly, the frequency of Th17 cells was reduced in patients after corticosteroid (CSs) and nonsteroidal anti-inflammatory drug (NSAIDs) therapy that were in remission phase. This new study shows a correlation between Th17 cells and disease progression; however, it remains unknown if Th17 cells are part of the pathogenic mechanism in development of AOSD.

An additional autoimmune disease in which the Th17 pathway has been implicated is childhood Henoch-Schönlein purpura (HSP), a common childhood systemic vasculitis in which increased frequency of peripheral Th17 cells and serum IL-17 levels occur [99]. There is also evidence from mice suggesting that Th17 cells contribute to autoimmune myocarditis development [100–102].

## 9. Conclusion

Substantial progress in understanding Th17 development and the effects of IL-17 signaling in immune responses has revealed high potential for targeting this pathway in immune pathologies. The ability to target or redirect T-cell lineage development can greatly ameliorate autoinflammation in preclinical models. However, a better understanding of the cytokines involved in the induction of Th17 differentiation and the balance between Th17 versus Treg and Th1 development during infection or autoimmunity requires further investigation. Finally, linking relevant animal disease models with clinical studies could improve our understanding of how these cells contribute to disease pathogenesis. 

## Figures and Tables

**Table 1:** Preclinical mouse models showing a role for Th17 cells and the IL-17 pathway in autoimmunity.

Disease	Model	Role of Th17	Reference
Rheumatoid arthritis	Collagen-induced arthritis (CIA)	IL-23 (p19 subunit) KO mice have less Th17 cells and reduced disease severity	[35]
	Collagen-induced arthritis (CIA)	IL-17A KO mice have reduced disease incidence and severity	[36]
	IL-6R subunit gp130 (Y759F) mutation	Enforced IL-6 expression enhances disease progression in an IL-17A-dependent manner	[37]
	SKG-dominant active ZAP70 mutant	IL-17A KO mice are resistant to arthritis	[39]
	HTLV-I Tg mice	IL-17A KO mice are resistant to arthritis	[41]

Inflammatory Bowel disease	Adoptive transfer of CD4^+^CD45RB^hi^ cells to lymphopenic mice	IL-17A KO CD4^+^ T cells accelerated T-cell-mediated intestinal damage	[42]
	Adoptive transfer of CD4^+^CD45RB^hi^ cells to lymphopenic mice	IL-17A, IL-17F, or IL-22 KO T-cells-induced colitis equally compared to WT	[43]
	Dextran sodium sulfate (DSS)	IL-17F KO or IL-17A KO or mice treated with an anti-IL-17A antibody showed severe weight loss and colonic epithelial damage	[44, 45]
	Dextran sodium sulfate (DSS)	IL-17A KO showed substantially reduced colitis based on both clinical score and mortality	[46]

	Topical application of imiquimod (IMQ)	Dermatitis is blocked in IL-23R KO and IL-17R KO mice	[53]
Psoriasis	IL-23 injection	IL-6-dependent accumulation of Th17 cells in psoriatic skin, dermatitis was greatly reduced in IL-22 KO or IL-17A KO mice	[55, 56]
	K5.Stat3C transgenic mice	Constitutively express activated Stat3 within keratinocytes promotes Th17 cells, and anti-IL-23 was shown to block epidermal hyperplasia; however, anti-IL-17 had only partial effect	[57]

Type 1 diabetes	RIP-OVA mice (mice expressing OVA peptide in pancreatic beta islet cells )	IL-17 producing CD8^+^ T cells (Tc17) cause diabetes in an IL-17A- and IL-17F-dependent manner when adoptively transferred to RIP-OVA mice	[59]
	Nonobese diabetes (NOD)	Anti-IL-17A antibodies inhibit diabetes during the effector phase of disease (at 10 weeks of age) but not during the initiation of disease (mice less than 5 weeks of age)	[60]

**Table 2:** Clinical aspect of therapies targeting the IL-17 pathway.

Disease	Sera	Biopsies	Cell type	SNPs or mutations	Therapy targeting Th17 pathway	Reference
Multiple sclerosis		IL-17A mRNA is detected in cerebrospinal fluid mononuclear cells	Myelin-reactive Th17^+^ cells are enriched and express high IL-22 and IFN*γ*		Anti-p40 subunit of IL-12/23 (ustekinumab) (no effect)	[73–75]
Rheumatoid arthritis	IL-17 ↑	IL-17 ↑	Synoviocytes express IL-1b and IL-6 in response to IL-17		Anti-IL-6R (Tocilizumab); IL-1 receptor antagonist (anakinra); anti-IL17 (LY2439821, others)	[69, 72, 76–78]
Systemic lupus erythematosus	IL-17 ↑	IL-17 ↑				[18, 70, 71]
Psoriasis		IL-17A, IL-22, and IL-23 ↑	CD8^+^ T cells secreting IL-17 and IL-22 (Tc17 and Tc22, respectively)	IL-23 pathway	Anti-p40 subunit of IL-12 and IL-23 (ustekinumab)	[79–82]
Inflammatory Bowel disease				IL-23 pathway		[83]
Crohn's disease		IL-17^+^ (ROR*γ*T^+^ IL-23R^+^ and CCR6^+^cells) ↑	Th17 (CD161^+^CD4^+^ T cells)		Anti-IL-6R (Tocilizumab); anti-p40 subunit of IL-12/23 (ustekinumab)	[76, 84–86]
